# Catastrophic failure of a titanium locking plate in a proximal humeral fracture: case report and literature review

**DOI:** 10.1186/s12891-022-05931-4

**Published:** 2022-11-05

**Authors:** Yan-Shiang Lian, Chang-Hung Huang, Min-Yao Chuang

**Affiliations:** 1 Department of Orthopaedic Surgery, MacKay Memorial Hospital, 10449 Taipei, Taiwan; 2 Department of Medical Research, Biomechanics Research Laboratory, MacKay Memorial Hospital, 25160 New Taipei City, Taiwan; 3 School of Dentistry, National Yang Ming Chiao Tung University, 11221 Taipei, Taiwan; 4 Department of Physical Therapy and Assistive Technology, National Yang Ming Chiao Tung University, 11221 Taipei, Taiwan

**Keywords:** Angular stable locking plate, Case report, Fatigue failure, Plate failure, Proximal humeral fracture, Torsion stress

## Abstract

**Background:**

Angular stable locking plates have shown good clinical results in treating proximal humeral fractures, but complications are not uncommon. This study reported a rare case of catastrophic failure of a titanium locking plate. A retrieval analysis of the implants was performed using an optic microscope and a scanning electron microscope.

**Case presentation:**

A 69-year-old male reported a right proximal humeral fracture at the surgical neck and was treated by open reduction and internal fixation with a locking plate system. Ninety-six days after surgery, the patient came to clinic for acute local pain over the shoulder without any trauma. The radiographs showed a complete breakage of the implant accompanying displaced fracture. Revision surgery was performed to restabilize the fracture with a longer locking plate. The follow-up radiographs at 9 months showed complete union of the bone fracture.

**Conclusions:**

From the retrieval analysis, repetitive torsion loads on the vulnerable area of the implant are assumed to cause this catastrophic event. It is recommended that adequate activity restriction, such as reaching, be undertaken to avoid this rare complication. Current study also provides contributive information for the modification of plate design and pre-operative planning for device configuration to improve the success rate of locking plate fixation.

## Background

Proximal humeral fractures are some of the most frequent fractures in the elderly population [1, 2, 3, 4, 5]. Due to the aging population, their incidence has increased nearly threefold in the past 30 years [5]. Advent of locking plate fixation has led to a dramatic increase in open reduction and internal fixation (ORIF) of proximal humerus fractures. The anatomy of the proximal humerus seems ideal for the application of this technology given the displacing forces of the rotator cuff, the tendency for varus displacement in a large proportion of fractures, the relatively osteopenic bone in the humeral head, and the limited surgical access to the posteromedial calcar. Clinical studies of locking plates for treating proximal humeral fractures for functional recovery have shown good to excellent results. However, this procedure is technically demanding and has a high rate of complications [6, 7, 8, 9]. Common complications include intra-articular screw penetration (9.5%), varus collapse (6.8%), subacromial impingement (5.0%), avascular necrosis of the humeral head (4.6%), adhesive capsulitis (4.0%), nonunion (1.5%), and deep infection (1.4%). Plate breakage is a rare but catastrophic complication that inevitably leads to revision surgery. Here, we report a case of titanium locking plate breakage in a proximal humeral fracture in our series (one of 713 cases at one institution over an 8-year period from 2011 to 2019). Those patients with 3- or 4-part complex fractures treated with reverse shoulder prosthesis or hemiarthroplasty were not included [10]. In addition, a retrieval analysis of the hardware was performed using an optical microscope, and the detailed fracture surfaces were observed by scanning electron microscopy.

## Case presentation

**Fig. 1:**
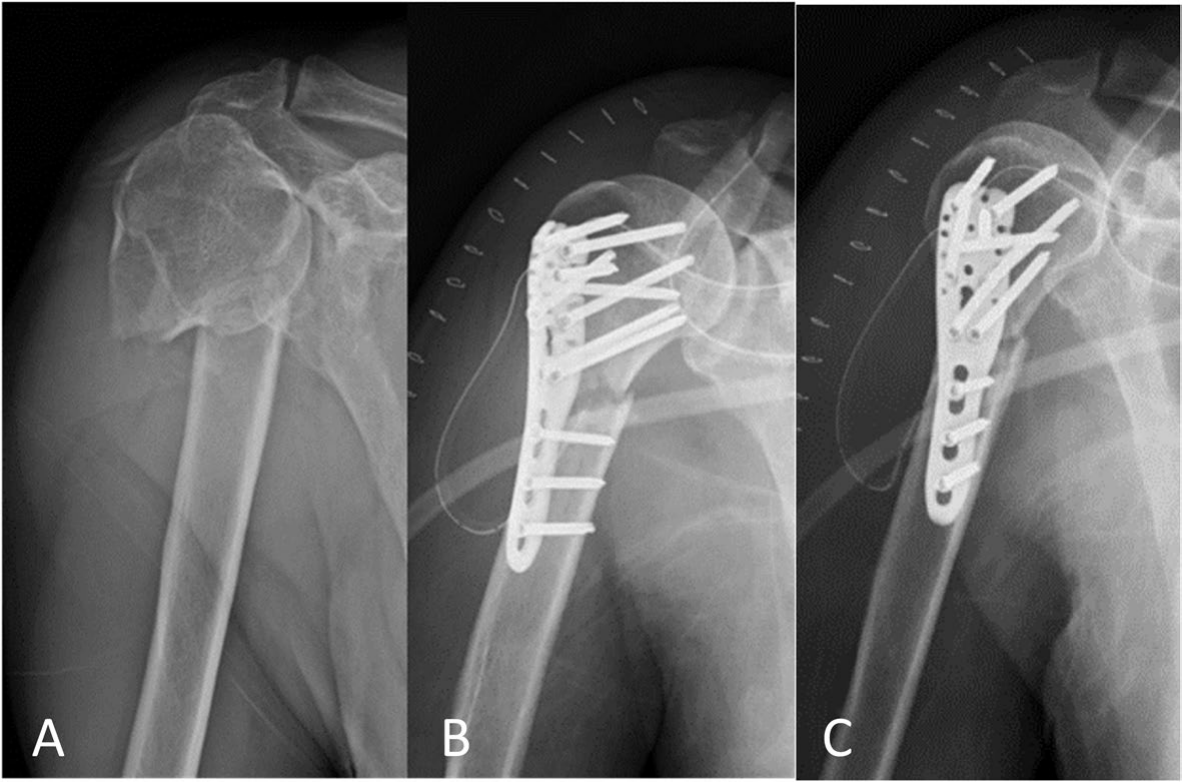
A displaced two-part proximal humeral fracture in a 69-year-old male patient. **A** Anterio-posterior radiograph reveals a displaced and comminuted fracture at the humeral surgical neck. **B** and **C** Anterior- posterior radiograph after surgery shows satisfactory reduction and fixation with a PHILOS plate

A retired 69-year-old male (body mass index: 18.9) reported a highly displaced two-part proximal humeral fracture at the surgical neck: a 1.1.A.3 fracture according to the AO classification or a two-part fracture according to the Neer classification (Fig. 1A). The patient was informed that data from the research would be submitted for publication, and gave his consent. Approval was also given by the institutional review board (IRB).

**Fig. 2:**
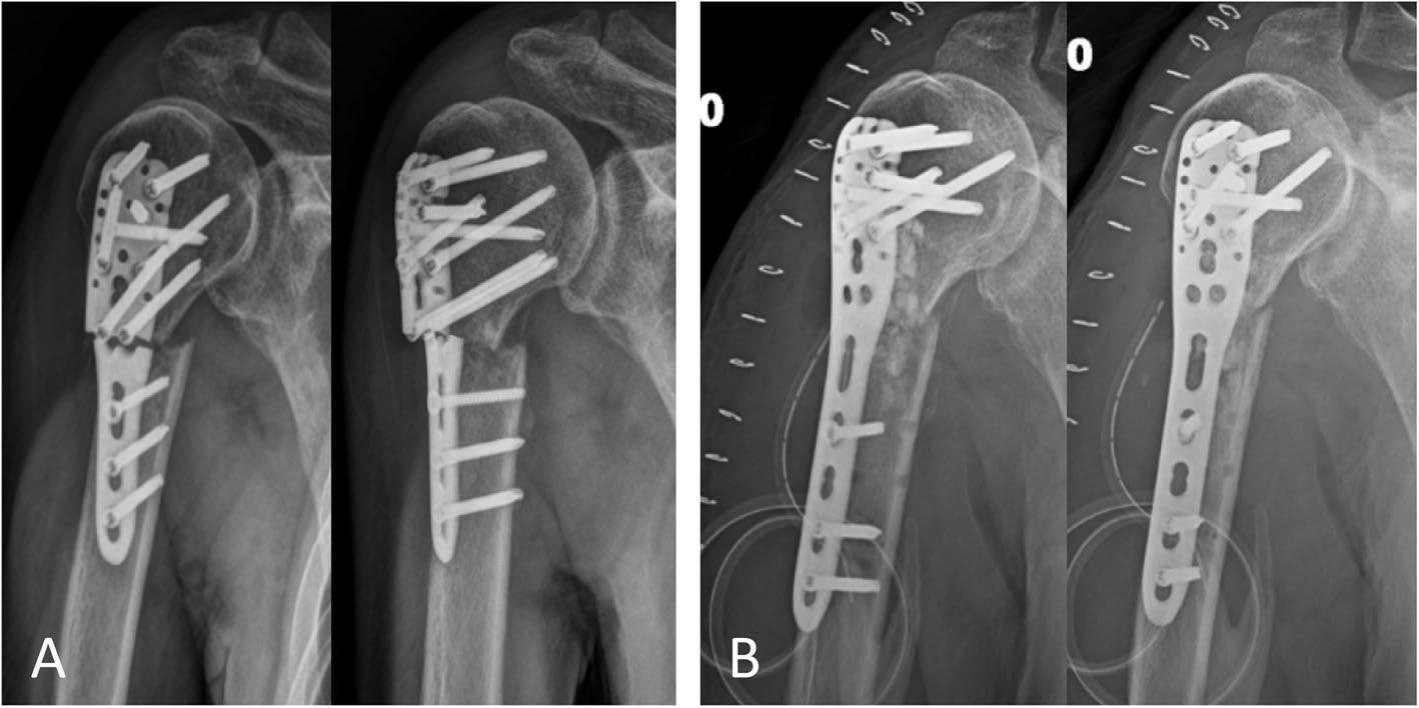
**A** Ninety-six days after surgery, a follow-up radiograph reveals breakage of the plate (90 mm in length) near fracture site. **B** Revision surgery is performed with a longer plate (114 mm in length). Nine months following revision surgery, the radiograph shows good alignment and union of the fracture

The fracture was treated by ORIF with a PHILOS system (DePuy Synthes, Zuchwil, Switzerland), which was performed through a transdeltoid anterolateral approach (Fig. 1B and C). In our institution, indication conference would be launched every morning for traumatology case discussion. The implant we choose during preoperative planning was a standard three shaft-hole plate (441.901) with 90 mm long in length with adequate working length on either side of fracture side and the soft tissue dissection would be minimized. After surgery, as the post-operative rehabilitation protocol for proximal humeral fracture, the patient was immobilized with an arm sling for 4 weeks. Follow-up radiographs showed callus formation and stable fixation of the fractures. Thereafter, an active and active-assisted physiotherapy regimen was implemented, starting with pendulum exercises. The mobilization was gentle, and was dictated by the patient’s level of pain; it intensified after 6 weeks in that the movements were more active than assisted. No resistive exercises or rotational movements were not performed until the 6th week to avoid further translation of the fragments. Ninety-six days after surgery, the patient visited our outpatient clinic for acute local pain and complete loss of function over the shoulder without any trauma. The radiograph showed complete breakage of the plate near the fracture site at the level of the surgical humeral neck (Fig. 2A).

**Fig. 3:**
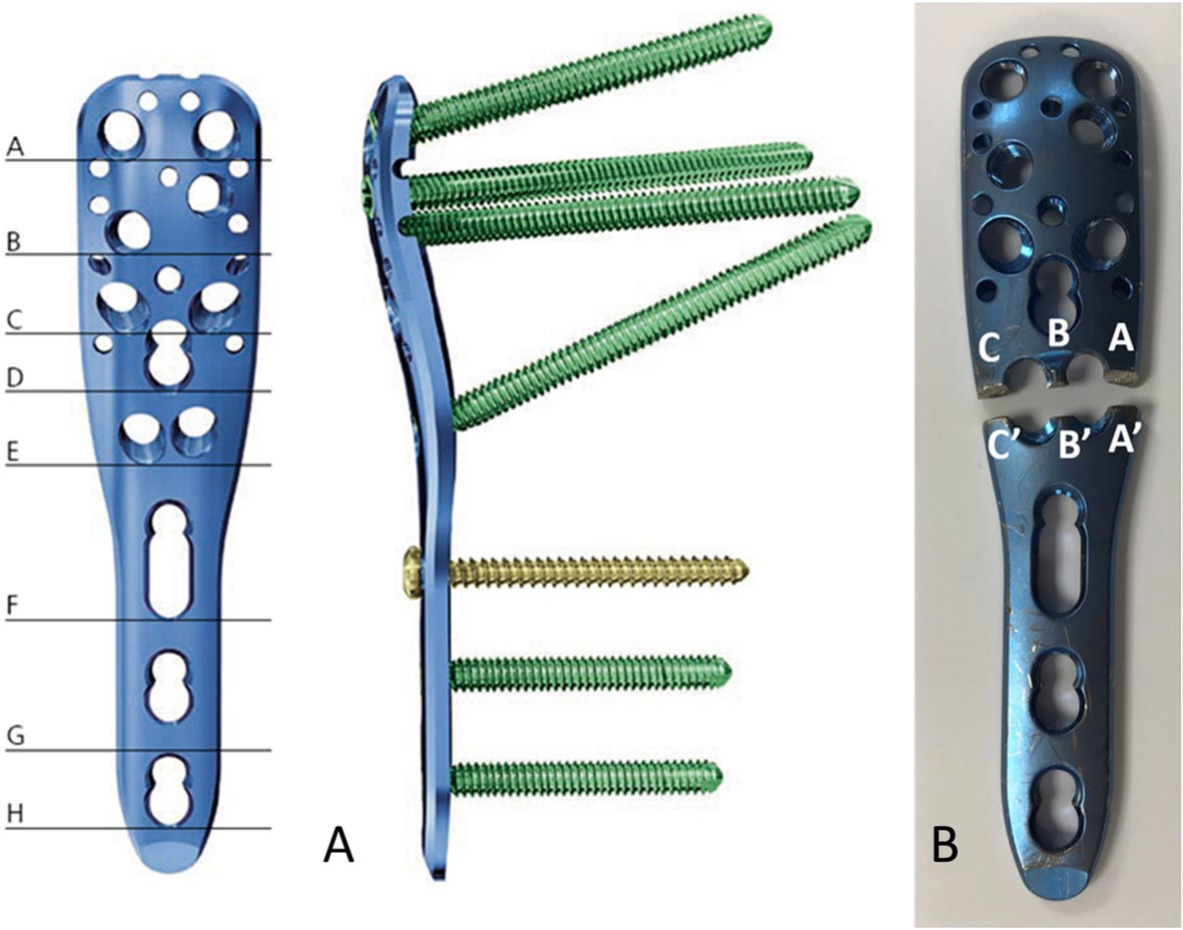
**A** Top view (left) and lateral view (right) of PHILOS system show the shape and thickness of the plate are transformed at areas between sections E and F. **B** Macroscopic inspection reveals that plate breakage occurs in the section of two adjacent screw holes (section E). These two photos are taken in our own Biomechanical laboratory

The patient underwent revision surgery 3 days after the clinic, during which, the plate was removed for retrieval analysis and the fracture non-union was observed. The displaced fracture was reduced and re-stabilized using a PHILOS system (DePuy Synthes, Zuchwil, Switzerland). A longer implant (441.903), five shaft-hole (114 mm long), was chosen, and screws were inserted bypassing the converting zone of the plate shape (between sections E and F in Fig. 3A). A demineralized bone matrix (Exactech®, Optecure®) and synthetic bone graft substitute (chronOS®, SYNTHES®) were used as adjuvants at the fracture site.

The patient was followed up 1 week, 1 month, 2 months, 3 months, 6 months, and 9 months after surgery. The latest follow-up radiographs at 9 months showed complete union of the bone fracture (Fig. 2B). Moreover, excellent functional results were demonstrated by visual analogue scale (0), short form 12 health survey (40), and constant shoulder score (79) evaluations.

Visual examination showed that the plate breakage was linked across the narrow bridge between the threaded screw holes, and spread from these holes to the edges of the plate (Fig. 3B). Analysis was performed on the cross-section of the breakage regions. After macroscopic inspection of the vulnerable points, microscopic examination by instruments was focused on the significant areas marked A, B, C, A’, B′, and C′ (Fig. 3B). Instrumental examination of the plate breakage surfaces was performed using an optical microscope (Leica MZ6, Leica Microskopie et System GmbH, Germany) and then observed using a scanning electron microscope (SEM) (Hitachi S-3500 N; Hitachi Science Systems, Ltd., Ibaraki, Japan).

**Fig. 4:**
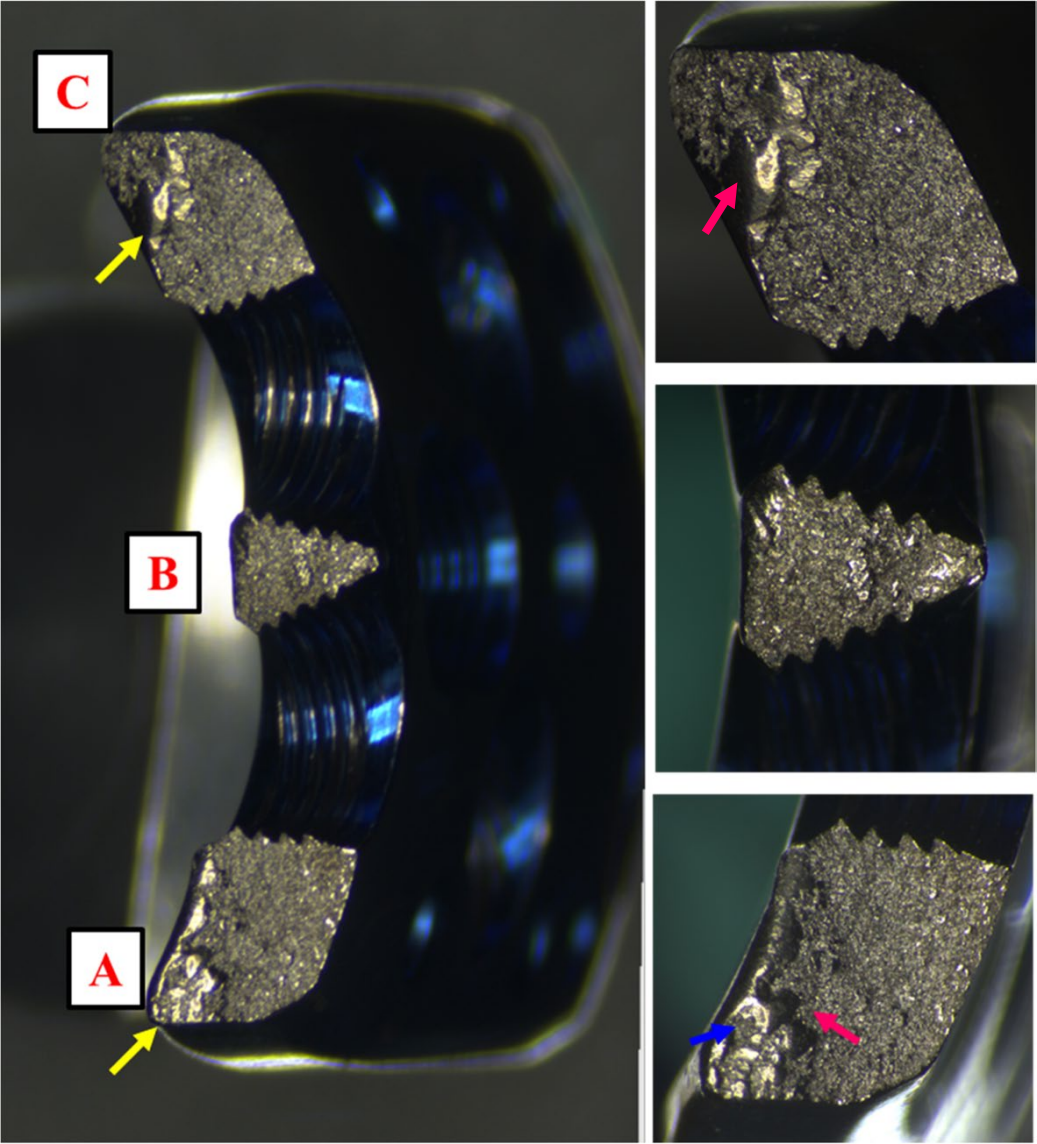
Optic microscopy images **A**, **B**, and **C** show a globular waved structure, characteristic of fatigue failure. Smooth areas (yellow arrows) suggest initial points of breakage. Asymmetrical ridges (red arrows) and notch (blue arrow) at A and C reveal metal destruction caused by torsional stress. These photos are taken in our own Biomechanical laboratory

The images of the optic microscopy analysis of the fracture areas (Fig. 4) revealed a globular waved structure, characteristic of fatigue failure. The fracture had been burnished peripherally in areas A and C, presumably as the initial points of breakage (yellow arrows). The asymmetrical ridges (red arrows) and notches (blue arrow) in areas A and C reveal metal destruction by torsion loads.

**Fig. 5:**
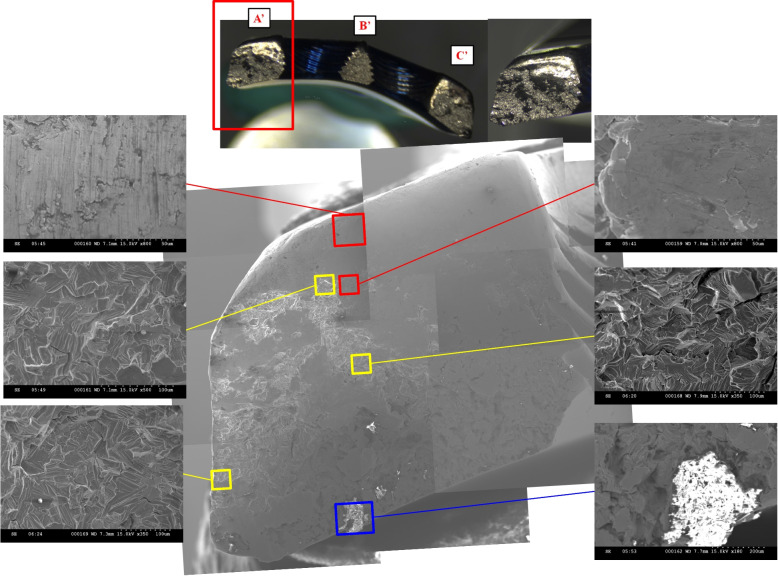
The SEM images of A’ marked by red frame (magnification × 800) show fatigue striations, suggesting crack origination. The morphology of the yellow frames (magnification × 350) represents a fatigue crack propagation. Ductile dimples at the blue frame (magnification × 180) indicate a dynamic fracture region. These photos are taken in our own Biomechanical laboratory

According to Griffith’s seminal work model and subsequent fracture mechanics studies describing the theory, stages of biomechanical behavior exist for implant breakage, including crack initiation, fatigue crack propagation, and a final dynamic fracture zone [8]. The SEM analysis of the fracture area A’ (Fig. 5), marked by a red frame, showed a wavy pattern with fatigue striation, suggesting a crack origination region. The morphology of the yellow frame was typical of the fatigue crack propagation region of the titanium alloys. Ductile dimples on the outer edge of the fracture area marked by the blue frame indicate a dynamic fracture region. No metallurgical or manufacturing defects, inclusions, or porosities were observed in any of the fracture areas.

## Discussion and conclusion

With the advent of locking screw technology, plating systems for treating proximal humeral fractures have been widely used in recent decades [9, 11]. Locking plates are introduced to improve fracture stability and are reliant on the bone-screw interface instead of the bone-plate interface [12]. The rigid fixation allowed by locking plates has been suggested to improve the initial mechanical stability and improve outcomes when compared to the conventional plate. Although clinical studies have shown good functional outcomes after locking plate systems for fractures of the proximal humerus, various studies have reported high rates of postoperative complications [6, 13]. Modern design features are used to minimize the possible complications of ORIF, such as varus displacement of the humeral head, loss of fixation, and nonunion, while plate breakage is a rare but severe complication [14]. Here, we report an unusual case of plate failure 96 days after surgery.

Many factors can determine the success of locking plates for the treatment of proximal humeral fractures, including initial stability and biomechanical performance after surgery [15], both of which can be affected by variables such as the bony fracture pattern, bone integrity, bone-plate offset, and number and spacing of screws. Other factors, such as material selection and design rationale of the plate as an implant shape, can also play important roles in the success rate of surgery.

Catastrophic breakage of the locking plate in the treatment of proximal humerus fractures is rare. The PHILOS system has nine locking screw holes in the proximal portion, which enables multiple directional screws to achieve an angular stable construct in the osteopenic bone or comminuted fracture. Given the unequal number of screw holes between the proximal and distal portions, the implant had an irregular shape around section E (Fig. 3A). To reduce soft tissue impingement after surgery, the plate was designed to match the anatomical contour of the proximal humerus, which led to the specific conformation shown in Fig. 3A. These design features of the plate shape which develop a stress riser around section E may contribute to the catastrophic failure of implants. It is also known that plate breakage often occurs at the level of cortical comminution.

Cyclic loading above the fatigue limit of titanium alloys can cause inevitable fracture of the plate after surgery. To prevent complications, a principle for optimizing the device configuration of the locking plate has been proposed [15]. One of the most important parameters regulating the device stiffness is the working length (the distance between the two innermost screws on either side of the fracture); a sufficient length can alleviate plate stress and promote fracture healing. Another cadaveric study concluded that the high initial stiffness of implants led to early loosening and failure of the implant-bone interface. Implants with better resilience can reduce the rigidity of the implant-bone interface by absorbing part of the energy. For the revision surgery in this case, preoperative planning of the length of the plate and the position of the screw for the surgical neck fracture are essential to achieve optimal configuration to promote union of the fracture. We choose a longer plate to achieve sufficient working length on either side of fracture and offering more flexibility of osteosynthesis may ensure fracture healing and avoid catastrophic consequences. In addition, screw tightening near fractured humerus bones could induce a complex stress distribution, resulting in a decrease in the initial stability.

The PHILOS system (DePuy Synthes, Zuchwil, Switzerland) is the most commonly used and tested anatomical locking plate system for proximal humeral fractures [16]. To the best of our knowledge, five catastrophic failures of locking plates treating proximal humeral fractures have been presented so far [17, 18]. All cases failed in areas around section E (Fig. 3A), as was observed in the current case. The main reasons for this may be the design features of the plate. The shape of the plate is irregular around section E in the top and lateral views in Fig. 3A. This design feature makes the areas around section E vulnerable to competitive stress. Shape modification of implants may decrease the incidence of fatigue failure.

Of the five catastrophic failures reported in the literature, only two clinical studies have analyzed these catastrophic events with regard to the design features of plate [17]. In these two studies, the plate was broken across the screw holes near section E (Fig. 3A), and the breakage region was the same as that reported in the present study. However, only the central region of the fractured plate was observed [17]. Therefore, the authors of the two studies assumed that the forces acting on the proximal fragment caused plate fracture around section E, and resulted in failure, predominately due to the bending moment. Although fatigue failure has been mentioned in these previous studies, it is difficult to identify the initiation point that results in plate breakage. A thorough observation of the entire fractured cross-sectional surface would be helpful to determine the initiation point and to help establish the weak point of the locking plate. Therefore, three regions of the cross-sectional surfaces of the plate were observed by optical microscopy to determine the possible weak point, and a further detailed observation was performed using SEM. In this fractured plate, one central and two peripheral fractured cross-sectional areas were examined. We found a peripherally burnished appearance, asymmetrical ridges, and notches in areas A and C by optic microscopy (Fig. 4). Therefore, it was inferred that torsion force initiated the failure process from the peripheral areas of the implant. In addition, the morphology at area A’ (Fig. 5) by SEM, which indicated the initiation region, reinforced this assumption.

In the current research, the analysis of the fracture areas of implants by optical microscopy and SEM revealed a pattern of fatigue failure without metallurgical or manufacturing defects. Because the locking plate is a long and thin structure designed with screw holes on it, prestresses after screw tightening can cause concentrated stresses on the plate. The plate-screw system plays a critical role in providing the initial stability prior to healing of the fractured bones. The failure process could be initiated with minor cracks in the peripheral areas due to torsion loads. This finding from the current retrieval analysis can be used to modify the design feature to eliminate severe complications, such as plate fracture. In addition, different implant materials, such as carbon-fiber reinforced polyetheretherketone (CFR-PEEK), could improve the biomechanical performance of the locking system to diminish this sequela [19].

Loading transfer can also affect the initial stability after ORIF. Indeed, a biomechanical study has reported that a large external torsional force can be generated at the upper limb and shoulder joint when performing common daily activities. The authors measured shoulder joint loads during daily living, and instrumented telemeterized joint implants were used to obtain realistic forces and moments transferred inside the glenohumeral articulations. The authors mentioned that common activities of the upper limbs, such as steering with one hand and setting down a pot, generated higher contact forces than lifting a weight of 10 kg. During one-handed steering, the peak resultant force on the joint reached an average of 122.4% body weight (BW) combined with an average moment of 0.4% BW-m. Therefore, these activities produce significant torque on the locking plate, especially if the bony fracture has not healed well. From a biomechanical point of view, stress concentration is generated on the cross-sectional surface of a thin titanium plate (with screw holes), leading to subsequent fatigue failure.

Locking plate technology was developed to reduce complications, such as delayed union, nonunion, refracture, and infection, following fracture fixation using conventional plates and screws. The locking system controls the axial orientation of the screw to the plate, thereby enhancing the screw–plate–bone construct stability by creating a single-beam construct. Single-beam constructs are four times stronger than load-sharing beam constructs, where motion occurs between the individual components of the beam construct. In a cadaveric study, the average maximum load to failure for osteosynthesis was greater in locking plates than in conventional plates. (876 N versus 712 N). Although the locking system provides higher mechanical strength, several implant-related complications have been reported in the literature [17, 18, 20]. Indeed, given the lack of friction force between the bone-plate interface in the conventional plate and screw, the stability of the locking system relies on the bone-screw and screw-plate interface. Thus, the configuration of osteosynthesis and the design features of implants play important roles in the initial stability after surgery using locking plates and screws.

Several strategies have been proposed to overcome this catastrophic failure. In terms of surgical techniques, achieving a sufficient working length while applying the plate may be beneficial for bone healing and relieving stress on the plate. In terms of the implant design, it is recommended to average the thickness of the implant and modify the shape of the plate. In addition, it is essential to avoid excessive torsion load during rehabilitation after surgery as this may result in the catastrophic stress observed in the current case.

This study has several limitations. First, we conducted a meticulous retrieval analysis to observe the fractured surface of the failed plate, which helps to determine the possible reasons for such severe complications. However, further biomechanical analysis is necessary to detect the stress distribution over cross-sectional fractured surfaces. Furthermore, a well-organized experiment using plates of different lengths and various locking screw arrangements will be helpful in determining the initial stability of osteosynthesis to ensure fracture union after surgery. We are currently planning future biomechanics studies to address this issue.

From the current retrieval study, it is assumed that repetitive torsion stress of daily activities on the vulnerable area of the plate has the potential to result in this catastrophic complication. This research indicated that fatigue striations were discovered around the outer regions of the cross-sectional surfaces of the locking plate, and the patterns were mainly caused by torsional stress. It is recommended that adequate activity restriction, such as reaching, be undertaken to help avoid this rare catastrophic event. We also provide contributive information for the modification of plate design and pre-operative planning for device configuration to improve the success rate of locking plate fixation.

## Data Availability

The data analyzed in this study are available from the corresponding author on reasonable request. All data in this study are included in this published article. The manuscript, including related data and figures has not been previously published and is not under consideration elsewhere.

## Abbreviations

BW: Body Weight
CFR-PEEK: Carbon-Fiber Reinforced PolyEtherEtherKetone
IRB: Institutional Review Board
ORIF: Open Reduction and Internal Fixation
SEM: Scanning Electron Microscope
